# *IFI16* Expression Is Related to Selected Transcription Factors during B-Cell Differentiation

**DOI:** 10.1155/2015/747645

**Published:** 2015-06-22

**Authors:** Pier Paolo Piccaluga, Claudio Agostinelli, Fabio Fuligni, Simona Righi, Claudio Tripodo, Maria Carla Re, Alberto Clò, Anna Miserocchi, Silvia Morini, Marisa Gariglio, Gian Gaetano Ferri, Alberto Rinaldi-Ceroni, Ottavio Piccin, Marco De Andrea, Stefano A. Pileri, Santo Landolfo, Davide Gibellini

**Affiliations:** ^1^Department of Experimental, Diagnostic, and Specialty Medicine, Bologna University Medical School Unit of Hematopathology, S. Orsola Malpighi Hospital, 40138 Bologna, Italy; ^2^Department of Human Pathology, University of Palermo, 90127 Palermo, Italy; ^3^Department of Experimental, Diagnostic, and Specialty Medicine, Bologna University Medical School Unit of Microbiology, S. Orsola Malpighi Hospital, 40138 Bologna, Italy; ^4^Department of Clinical and Experimental Medicine, Medical School of Novara, 28100 Novara, Italy; ^5^Department of Experimental, Diagnostic, and Specialty Medicine, Bologna University Medical School Unit of Otolaryngology, S. Orsola Malpighi Hospital, 40138 Bologna, Italy; ^6^Department of Public Health and Microbiology, University of Turin, 10126 Turin, Italy; ^7^Department of Pathology and Diagnostic, University of Verona, 35124 Verona, Italy

## Abstract

The interferon-inducible DNA sensor IFI16 is involved in the modulation of cellular survival, proliferation, and differentiation. In the hematopoietic system, IFI16 is consistently expressed in the CD34+ stem cells and in peripheral blood lymphocytes; however, little is known regarding its regulation during maturation of B- and T-cells. We explored the role of IFI16 in normal B-cell subsets by analysing its expression and relationship with the major transcription factors involved in germinal center (GC) development and plasma-cell (PC) maturation. *IFI16* mRNA was differentially expressed in B-cell subsets with significant decrease in *IFI16* mRNA in GC and PCs with respect to naïve and memory subsets. *IFI16* mRNA expression is inversely correlated with a few master regulators of B-cell differentiation such as *BCL6, XBP1, POU2AF1*, and *BLIMP1*. In contrast, *IFI16* expression positively correlated with *STAT3, REL, SPIB, RELA, RELB, IRF4, STAT5B*, and *STAT5A*. ARACNE algorithm indicated a direct regulation of *IFI16* by *BCL6*, *STAT5B*, and *RELB*, whereas the relationship between *IFI16* and the other factors is modulated by intermediate factors. In addition, analysis of the CD40 signaling pathway showed that *IFI16* gene expression directly correlated with NF-*κ*B activation, indicating that IFI16 could be considered an upstream modulator of NF-*κ*B in human B-cells.

## 1. Introduction

The adaptive immune response mainly depends upon B- and T-cells that originate from hematopoietic stem cells [1]. B-cells mature in the bone marrow and are released into the peripheral blood to subsequently reach secondary lymphoid tissues. During the T-cell-dependent antibody response to exogenous antigens, B-cells undergo activation, forming the characteristic germinal center (GC) that is detectable in peripheral lymphoid tissues such as the spleen, tonsils, Peyer's patches, and lymph nodes [2, 3]. GC formation requires the interaction between costimulatory B-cell-surface receptors with specific ligands expressed on T helper cells and/or antigen-presenting cells. Antigen-activated B-cells undergo a progressive clonal expansion and differentiation into centroblasts in the dark zone of the GC where the process of somatic hypermutation leads to base-pair changes in the V(D)J region of rearranged genes that encode the Ig-variable region of immunoglobulin chains. Centroblasts then differentiate into centrocytes and migrate into the light zone of the GC where a strong process of selection eliminates by apoptosis the centrocytes presenting low-binding antibodies. Selected centrocytes can then differentiate into plasma cells (PCs), responsible for antibody production, or into memory cells [4].

Although the specific transcriptional program of naïve B-cell expansion and differentiation into PCs is not yet fully understood, several transcription factors are functional differentiation markers. Specifically, BCL6, PAX5, BLIMP1, NF-*κ*B, some STAT family members, and IFN-induced factors are required for specific differentiation steps as demonstrated in knockout mice, where the deletion of some of these genes induced the lack of specific B-cell subsets [5–12]. Recently, the IFN-induced factor IFI16 protein has been associated with B-cell dysfunction and autoimmune diseases suggesting a possible role for this protein in B-cell biology [13]. IFI16 is a major member of the IFN-inducible PYHIN protein family, which consists of human and mouse proteins that are characterized by their ability to induce interferon, nuclear localization, and hematopoietic expression [14]. The PYHIN proteins may, therefore, serve as receptors for microbial DNA, providing new insights into infectious diseases. IFI16 contains two specific stretches of 200 amino acids (HIN-200 domain), designated a and b domains, at the C-terminus, and the PYD domain at the N-terminus [14, 15]. The* IFI16* gene encodes three protein isoforms that are generated from the translation of three separate mRNAs, which are produced by alternative mRNA splicing [16–19]. In normal human bone marrow, IFI16 expression is detected in CD34+ hematopoietic stem cells and throughout differentiation into monocytes and lymphocytes; however,* IFI16* expression is downregulated when CD34+ hematopoietic stem cells differentiate into red cells, neutrophils, or eosinophils [17]. Several studies have demonstrated that IFI16 plays an important role in the modulation of cell proliferation, survival, and senescence. IFI16 negatively regulates the cell cycle through the binding and functional modulation of several molecules involved in cell cycle regulation such as p53, Rb, and p21 [15, 19–27]. In particular, IFI16 is associated with cell cycle arrest in G0/G1 and/or G2/M phases in some cell lineages [28, 29]. IFI16 overexpression is also related to apoptosis activation [30–32], and the slow dividing hematopoietic progenitor CD34+ cells exhibit an approximately 4-fold increase in IFI16 expression with respect to the fast-dividing subset of the hematopoietic progenitor CD34+ cells [33].

*IFI16* expression is deregulated in autoimmune diseases and primary cancers [23, 36]. Although* IFI16* expression can be regulated through treatment with many differentiation stimuli [37], IFI16 is primarily induced by interferon (IFN) types I and II, and its expression is related to specific IFNs and cell types [38]. Furthermore, IFI16 plays a direct role in IFN-*β*-mediated responses because it responds directly to intracellular exogenous DNA in a Toll-like receptor-independent fashion [39, 40]. Association with IFN activity indicates a possible involvement of IFI16 in some autoimmune diseases that feature high interferon levels. Moreover, a possible relationship between IFI16 and prolonged NF-*κ*b activation that affects B-cell survival and induces B-cell cycle derangement especially through the p53 pathway has been suggested [13]. However, there have been limited studies on IFI16 in lymphoma cell lines and multiple myeloma [41–43], and no data are available on normal B-cell subsets.

In this study, we focused our attention on IFI16 expression in normal B-cell subsets. Our goal was to assess the* IFI16* expression patterns and their possible relationships with the most relevant transcription factors controlling B-cell development.

## 2. Materials and Methods

### 2.1. Isolation and Characterization of B-Cell Subsets

Whole blood samples were collected from healthy blood donors through venipuncture in EDTA-containing tubes after providing informed consent following the Helsinki declaration. Peripheral blood mononuclear cells (PBMCs) were separated using a Ficoll gradient (Ficoll-Hystopaque, Pharmacia, Uppsala, Sweden). Naïve and memory B-cells were purified from healthy donor blood using a naïve B-cell isolation kit (StemCell, Grenoble, France) or a memory B-cell isolation kit (Miltenyi, Auburn, CA, USA), respectively, following the manufacturers' instructions. The naïve and memory B-cells were analyzed using flow cytometry after the isolation procedure to determine the purity percentage of these B-cell subsets. CD19^+^/CD27^+^ and CD19^+^/CD27^−^ B-cells consisted of >95% in purified memory and naïve B-cells, respectively.

### 2.2. Gene Expression Analyses

We analyzed the gene expression profile (GEP) data that were previously generated and reported from different subsets of human B-cells [44, 45]. Briefly, we analyzed the GEP data from 25 samples of normal B-lymphocytes (naïve cells, *N* = 5; germinal center cells, *N* = 10; memory cells, *N* = 5; plasma cells, *N* = 5). All data were obtained by using the Affymetrix HG-U133 2.0 plus microarray (Affymetrix, Inc. http://www.affymetrix.com/support/index.affx) and are available at http://www.ncbi.nlm.nih.gov/projects/geo/. For further technical details, see [45]. In particular, we focused on the expression of* IFI16*, which was identified using three different probe sets with the HG-U133 2.0 plus GeneChip (206332_s_at; 208966_x_at; and 208965_s_at). The mean values from the three probes were used for the analyses. Because the GEP data were derived from different experiments, adequate normalization quality control was performed as previously reported and verified through box plot and MA plot consistency analyses [46]. GEP analyses were performed using GeneSpring GX 12.0 Software (Agilent Technologies, Santa Clara, CA, USA) [44–46].

To assess the effects of CD40 signaling on* IFI16* gene expression, we analyzed the previously reported GEP data [47]. Briefly, these data were originally generated using retroviral transduction to induce CD40 signaling in Burkitt lymphoma cell lines [47]. The CEL files that were originally available at GEO dataset GSE2350 were analyzed were analyzed using GeneSpring GX 12.0. Supervised analysis was conducted as previously reported [45] using a *p* value and fold change cut-off of 0.05 and ≥2, respectively, and a multiple test correction according to Benjamini-Hochberg was adopted [45]. IFI16 interaction with master B-cell regulators (selected based on their relevance for mature B-cell development according to the current literature [4], such as* BLIMP1*,* BCL6*,* MTA3*,* PAX5*,* IRF4*,* IRF8*,* XBP1*,* RELA*,* RELB*,* REL*, Oct-binding factor 1 (*POU2AF1*),* SPIB*,* BACH2*,* STAT3*,* STAT5A,* and* STAT5B*) was evaluated by calculating the coefficient of determination (*R* ^2^) and the Pearson correlation (see the Statistical Analyses section). Only the genes showing a *R* ^2^ > 0.5 and a Pearson correlation significance with *p* value <0.01 were selected for further analysis. The selected genes were then inferred by applying the ARACNe algorithm. To maximize the statistical significance, we referred to a large dataset of human normal and neoplastic B-cells as well as human B-cell lines that has been reported previously [45, 48] and is available at GEO datasets GSE2350 and GSE12195 ARACNe was performed using geWorkbench software, with bootstrapping, at a *p* value threshold of <0.01 before correction for multiple testing [45, 48–51]. PCs were eventually excluded from the analyses between* IFI16-BCL6*, and* IFI16-IRF4*. In fact, based on our analysis,* IFI16* expression was suppressed by other molecules in PCs, making them unsuitable for an appropriate evaluation of the relations between* IFI16*-*BCL6* and* IFI16*-*IRF4*. For the NF-*κ*B pathway analysis we also studied the expression of well-known transcriptional targets, such as* BCL2*,* CCND2*,* CCR7*,* CFLAR*,* IL2*,* IRF4*, and* NFKBIA*, which have been previously used to define NF-*κ*B activation by GEP [52]. In particular (i) we studied their expression levels (normalized microarray data), (ii) we studied the mean value of their expression in each sample (referred to as “NF-*κ*B signature”), and finally (iii) we divided the analyzed samples into two groups (NF-*κ*B+ and NF-*κ*B−) based on whether the expression levels of the NF-*κ*B signature were above or below the median value.

### 2.3. Quantitative RT-PCR

IFI16 mRNA expression analysis was performed through quantitative real-time RT-PCR (qRT-PCR) on RNA extracted from peripheral blood purified B-cell subsets achieved from three healthy donors (*n* = 3, two men and one woman, age between 32 and 36 years). Total RNA was extracted from purified B-cell subsets using the High Pure RNA isolation kit (Roche, Mannheim, Germany) and stored at −80°C. Then, total RNA was reverse transcribed and amplified employing the Quantitect SYBR Green RT-PCR kit (Qiagen, Hilden, Germany) following the manufacturer's procedure. In brief, SYBR green real-time PCR assay was performed in a 20 *μ*L PCR mixture volume consisting of 10 *μ*L of 2X Quantitect SYBR green RT-PCR Master Mix (Qiagen), containing HotStarTaq DNA polymerase, 400 nM of each oligonucleotide primer, 0.2 *μ*L of 100X QuantiTect RT Mix (containing Omniscript and Sensiscript reverse transcriptases), and 100 ng of extracted RNA. The IFI16 primer sequences [53] were as follows: IFI16F: 5′-ACAAACCCGAGAAACAATGACC-3′, nt 1464–1485, (RNArefseq NM_005531.2) and IFI16R: 5′-GCATCTGAGGAGTCCGAAGA-3′ nt 1584–1565, (RNArefseq NM_005531.2). These primers amplified a 121-bp region detectable in the different IFI16 isoforms. The amplification was performed in a LightCycler (Roche, Mannheim, Germany) with an RT step (1 cycle at 50°C for 20 min) followed by the initial activation of HotStarTaq DNA Polymerase at 95°C for 15 min and 40 cycles in three steps: 94°C for 10 s; 60°C for 15 s; and 72°C for 30 s. The LightCycler 5.3.2 software determined the quantification cycle (Cq). The* IFI16* amplicons were checked through a melting analysis, and a single sharp peak was detected (Tm = 82.5°C). The relative* IFI16* mRNA expression levels and the normalization to ribosomal* 18S RNA* were calculated using the ΔΔC_t_ method as described previously [34, 35, 54].

### 2.4. Protein Analyses

IFI16 protein expression was determined in naïve and memory B-cells isolated from healthy donor peripheral blood through flow cytometry and western blot procedures. For flow cytometry, purified B-cell subsets were achieved from five healthy donors (*n* = 5, three men and two women, age between 30 and 36 years). Purified naïve or memory B-cells were fixed in 1% PF for 30 minutes at room temperature. After several washes in PBS, the cells were treated for 45 minutes with 0.2% saponin (Sigma, St. Louis, MO, USA)/PBS and then with a rabbit anti-IFI16 antibody (Sigma) or, as a negative control, with a rabbit anti-HIV-1 p24 antibody (Diatheva, Pesaro, Italy) diluted 1 : 40 in 0.2% saponin/PBS for 30 minutes at room temperature. After washing with PBS, the samples were stained with FITC-anti-rabbit IgG (Dako, Copenhagen, Denmark) diluted 1 : 100 in 0.2% saponin/PBS for 30 minutes at room temperature. The samples were analyzed using FACSCalibur flow cytometry (Becton-Dickinson) and CellQuest software (Becton-Dickinson).

Western blot analysis was performed on purified naïve or memory B-cells achieved from healthy donors (*n* = 3; two men and one woman, age between 32 and 36 years). The cells were lysed in Laemmli buffer and equivalent amounts of proteins per sample were subjected to electrophoresis on a gradient 4–12% sodium dodecyl sulfate- (SDS-) acrylamide precast gel (Thermo Scientific, Waltham, MA, USA). The gel was then blotted onto a nitrocellulose membrane, and equal loading of protein in each lane was checked by staining of the blot membrane with 0.1% Ponceau S followed by destaining with distilled water. IFI16 protein detection was performed using rabbit anti-IFI16 antibody (Sigma, St. Louis, MO, USA) at 1 : 200 dilution, in blocking buffer (3% BSA in TBS/0.05% Tween 20) for 2 hours and after several washes in TBS/0.05% Tween 20, the nitrocellulose membrane was treated with alkaline phosphatase-conjugated goat anti-rabbit IgG (Sigma) at 1 : 1000 dilution in blocking buffer for 2 hours. Immunoreactive bands were visualized with BCIP/NBT solution (Sigma). As control, tubulin protein was detected by mouse anti-tubulin monoclonal antibody (mAb; Sigma) at 1 : 200 dilution and revealed by goat anti-mouse IgG (Sigma) at 1 : 1000.

Double immunofluorescence labeling on processed paraffin sections of reactive lymph nodes was performed as described previously [55]. Pressure cooked, microwave-treated slides were incubated for 1 hour with the following mixtures of antibodies directed to (i) CD20 (mouse mAb, clone L26, dilution 1 : 200, Dako) and IFI16 (rabbit antibody, dilution 1 : 30; Sigma); (ii) BCL6 (mouse mAb, clone PG-B6p, dilution 1 : 30, M7211, Dako) and IFI16; (iii) PRDM1/BLIMP1 (mouse mAb, clone ROSI95G, dilution 1 : 10, kindly provided by Dr. Roncador, CNIO, Madrid) and IFI16; and (iv) CD138 (rabbit antibody, dilution 1 : 400, RB 9422-P, NeoMarkers) and anti-PRDM1/BLIMP1. The slides were then incubated in the dark for 1 hour with the appropriate fluorophore-conjugated secondary antibodies (Alexa Fluor 568-conjugated goat anti-mouse IgG2a, Invitrogen, Carlsbad, CA, USA, for CD20, and Alexa Fluor 488-conjugated goat anti-rabbit IgG, Invitrogen, for IFI16 and CD138, and Alexa Fluor 568, goat anti-mouse IgG1, Invitrogen for BCL6 and PRDM1/BLIMP1). The micrographs were obtained using an Olympus BX61 microscope equipped with an Olympus DP-70 digital camera; image acquisition, evaluation, and color balance were performed using Cell^∧^F software.

### 2.5. Statistical Analyses

Statistical analyses were performed with the StatView 5.0 software package (SAS Institute Inc, Cary, NC) and Wessa [56], Pearson Correlation (v1.0.3) in Free Statistics Software (v1.1.23-r6), Office for Research Development and Education, URL http://www.wessa.net/rwasp_correlation.wasp/. ANOVA, unpaired *t*-tests, and, when appropriate (specifically, when the sample size was less than 10 cases in at least 1 group), a nonparametric (Mann-Whitney) test were adopted for GEP data analyses for the comparison of* IFI16* expression in different subgroups. The limit of significance for all analyses was defined as *p* < 0.05; two-sided tests were used in all calculations. The possible relationship between the expression of* IFI16* and other genes (transcription factors regulating B-cell development) was calculated using Pearson's correlation method and linear regression analysis. Relations were regarded as significant for *R* ^2^ > 0.50 and Pearson correlation with a *p* value <0.01.

## 3. Results

### 3.1. Both* IFI16* mRNA and Protein Expression Are Downregulated during Germinal Centre Reaction and Plasma Cellular Differentiation

**Figure 1:**
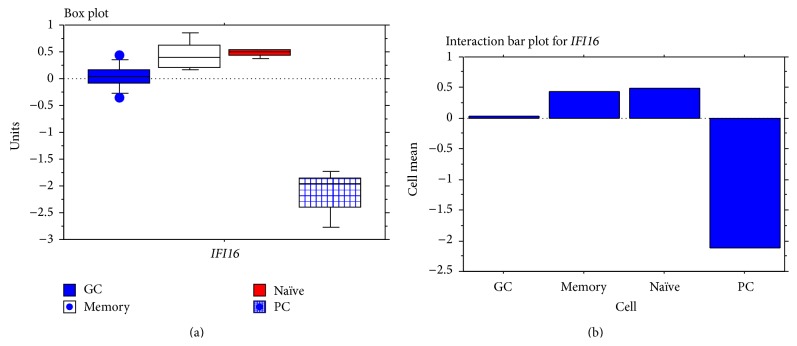
*IFI16* mRNA expression in normal B-cell subsets.* IFI16* mRNA expression evaluated by GEP and presented in box plot (a) and interaction bar plot (b). Naïve and memory B-cells showed a significantly higher* IFI16* mRNA level with respect to GC B-cells (naïve versus GC *p* = 0.0062; memory versus GC *p* = 0.0131; Mann-Whitney test) and PCs (naïve versus plasma cells *p* < 0.0001; memory versus PCs *p* < 0.0001; Mann-Whitney test) but no differences were noted between naïve and memory B-cell subsets (*p* = 0.78; Mann-Whitney test). GC cells showed a significant increase with respect to plasma cells (*p* < 0.0001; Mann-Whitney test).

To investigate whether IFI16 was modulated during B-cell functional differentiation, we studied* IFI16* gene and protein expression in normal B-cell subsets. IFI16 gene expression was analysed using data previously generated by DNA-microarrays [44, 45]. Naïve and memory B-cells displayed a significantly higher amount of IFI16 mRNA than GC B-cells (naïve versus GC *p* = 0.0062; memory versus GC *p* = 0.0131) or plasma cells (*p* < 0.0001; Figure 1 and Table 1). Conversely, no significant differences in* IFI16* mRNA expression between naïve and memory B-cells were detected (*p* = 0.78), although a consistent variation between individuals was observed in memory B-cells (Figure 1). Finally, GC B-cells exhibited significantly higher* IFI16* mRNA levels than PCs (*p* < 0.0001; Figure 1 and Table 1).

**Figure 2:**
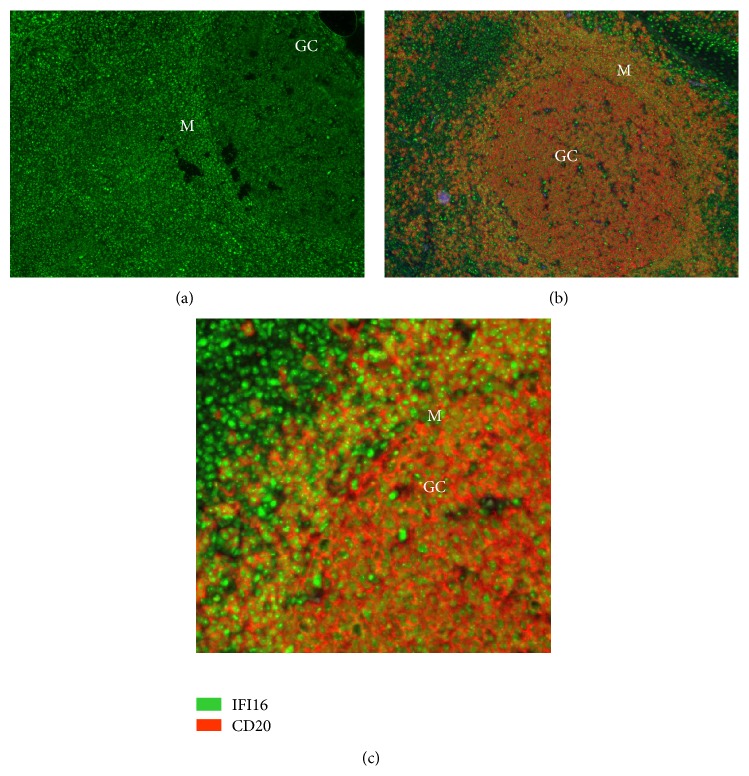
*IFI16 protein expression in normal lymphoid tissues*. (a) IFI16 staining of GC and mantle zones. (b) Double staining with CD20 and IFI16 antibodies. IFI16 protein expression in reactive lymphoid follicles; the expression pattern was largely restricted to B-lymphocytes of the mantle zone (M) and germinal centers (GC). (c) Particular of panel (b). Higher fluorescence in B-cells of the mantle zone (M) indicates a higher expression in this compartment compared with GC cells. These micrographs were obtained using an Olympus BX61 microscope equipped with an Olympus DP70 digital camera (magnification 100–400x); image acquisition, evaluation, and color balance were performed with Cell^∧^F software.

As IFI16 can also be regulated posttranscriptionally [57], its expression, at the protein level, was analyzed in lymph nodes using an indirect immunofluorescence assay. Double staining with antibodies directed against IFI16 and the B-cell marker CD20 clearly demonstrated IFI16 protein in the lymph node compartments. As expected, there was strong CD20 staining in the germinal center and in the mantle zone, and IFI16 was detectable inside the nucleus of CD20 positive B-cells (Figures 2(a)–2(c)). In accordance with the gene expression results, IFI16 staining was clearly brighter in the mantle zones, which are normally populated by naïve and memory cells, than in the GCs, which are basically composed of centroblasts and centrocytes (Figure 2(c)).

**Figure 3:**
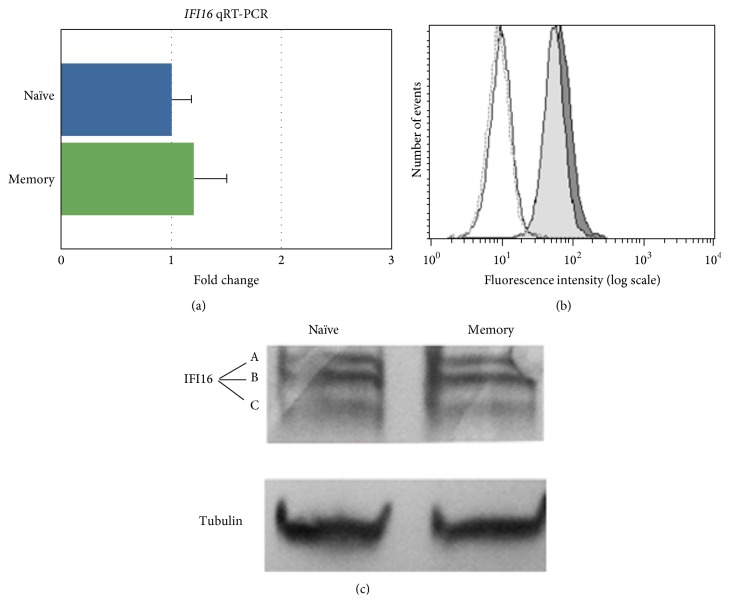
*IFI16* protein and mRNA expression analysis in naïve and memory B-cells purified from peripheral blood. In (a), IFI16 mRNA levels were determined using qRT-PCR. The IFI16 mRNA expression relative quantification was calculated with the ΔΔC_t_ method [34, 35]. The results are shown for the naïve B-cell subset relative to the memory B subset. The data represent the mean (±SD) of three independent experiments performed in duplicate. In (b), flow cytometry analysis of intracellular IFI16 protein was performed in naïve and memory B-cell subsets purified from peripheral blood. Naïve (light grey histogram) and memory (grey histogram) B-cells were stained by indirect immunofluorescence with a rabbit anti-IFI16 antibody (1 : 40 in 0.2% saponin/PBS) and, subsequently, with a FITC-conjugated sheep anti-rabbit IgG (1 : 100 in 0.2% saponin/PBS). The white histograms are the negative controls (dotted line, memory cells, solid line, and naïve cells) represented by naïve and memory B-cells stained with indirect immunofluorescence with a rabbit anti-HIV-1 p24 antibody (1 : 40 in 0.2% saponin/PBS) and, subsequently, with a FITC-conjugated sheep anti-rabbit IgG (1 : 100 in 0.2% saponin/PBS). A representative experiment is shown. In (c), western blot analysis of protein extract from peripheral blood memory and naïve B-cell subsets (*n* = 3). Cell lysates were separated by gel electrophoresis and transferred to nitrocellulose membrane. The proteins were probed with rabbit anti-IFI16 polyclonal antibody and then incubated with an AP-conjugated anti-rabbit IgG and detected by colorimetric procedure. Tubulin protein was assayed as control. IFI16 A, B, and C isoform proteins were expressed similarly in memory and naïve B-cell subsets. A representative experiment is shown.

Because the mantle zones can be quite heterogeneous in their composition, we also evaluated IFI16 expression in naïve and memory B-cell subsets purified from healthy donor peripheral blood samples using immunomagnetic procedures. Using flow cytometry, slightly higher IFI16 expression levels in memory cells compared with naïve B-cells were detected (Figure 3), although the difference was not significant. Western blot analysis of protein extracts from peripheral blood naïve and memory B-cell subsets demonstrated the presence of the three IFI16 isoforms generated by alternative splicing (Figure 3). Consistently, qPCR assays demonstrated that the* IFI16* mRNA content was similar in peripheral blood naïve and memory B-cell subsets (Figure 3), thus confirming the lymph node observations. Taken together, these results indicated that IFI16 expression was modulated throughout B-cell differentiation with a progressive downregulation during the GC reaction and differentiation to PCs.

### 3.2. *IFI16* Expression Correlates with Select Transcription Factors

**Figure 4:**
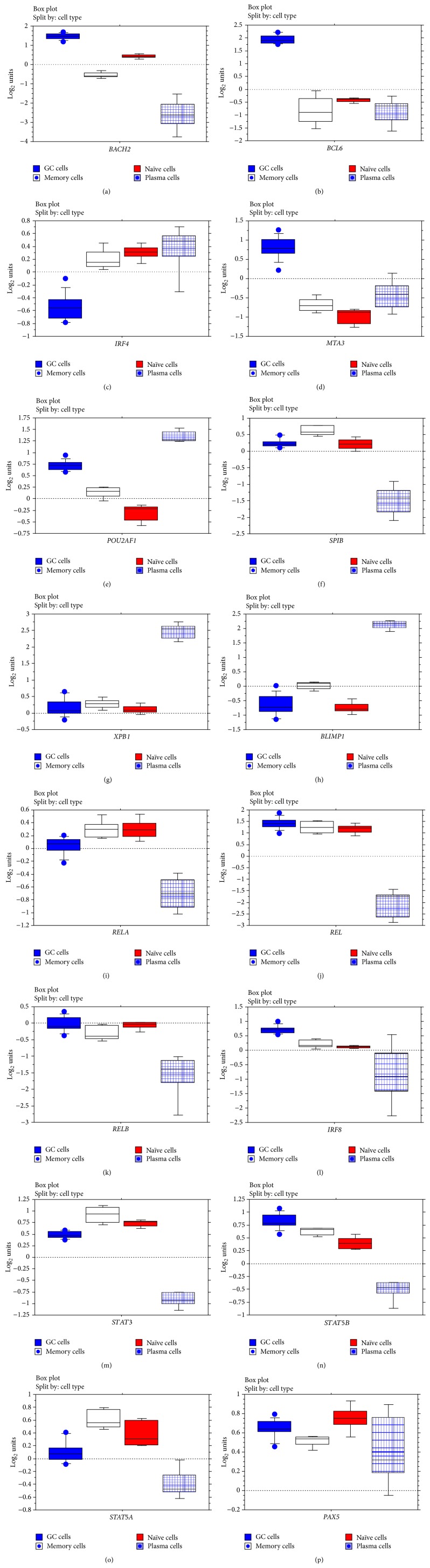
Gene expression levels of B-cell development associated transcription factors in normal B-lymphocytes. Normalized mRNA expression evaluated by GEP is presented in box plots.

**Figure 5:**
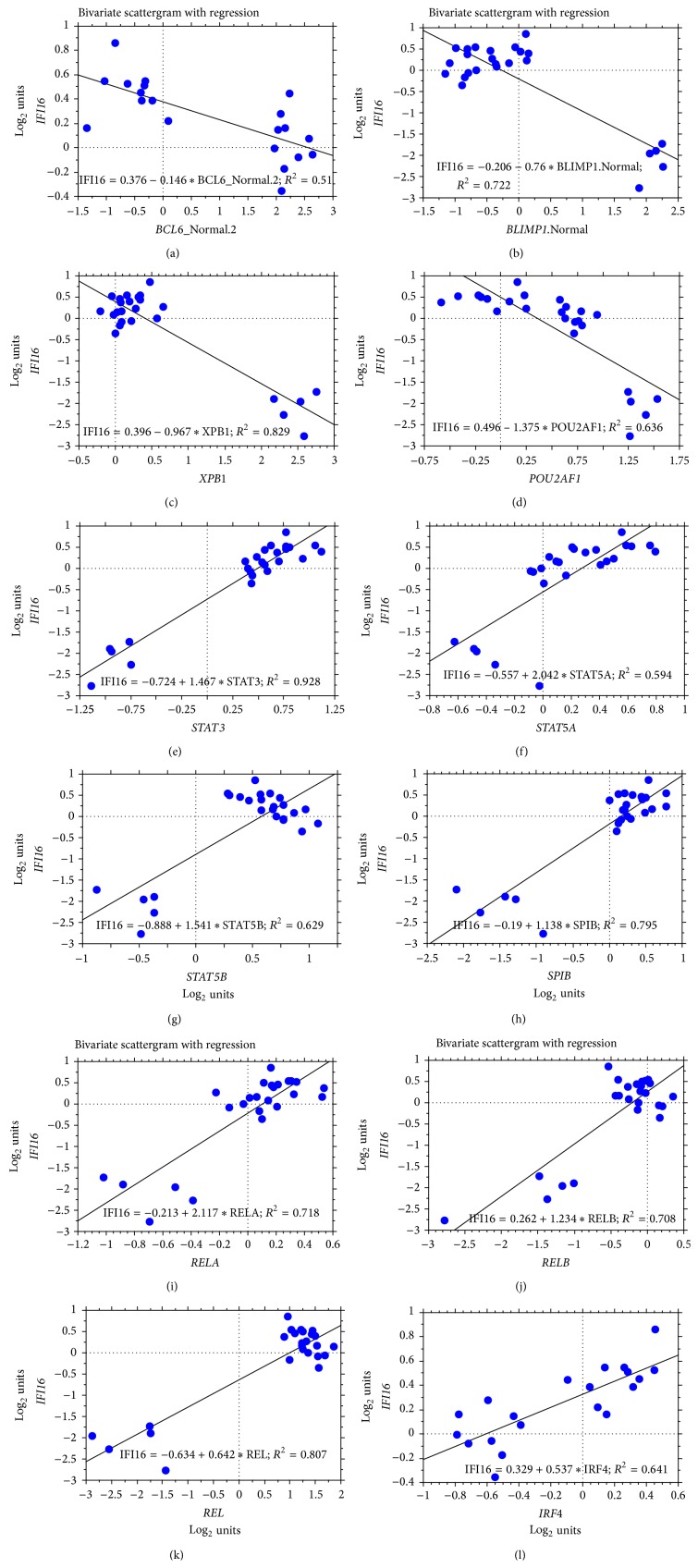
The correlation between* IFI16* and selected transcription factors was determined by GEP in normal B-cell subsets. The data are shown in a bivariate scattergram with a regression line.* IFI16* is plotted on the *y*-axes, while* BCL6*,* BLIMP1*,* XBP1*,* POU2AF1*,* STAT3*,* STAT5A*,* STAT5B*,* SPIB*,* RELA*,* RELB*,* REL*, and* IRF4* are plotted on the *x*-axes in panels (a) to (l), respectively. Note that plasma cells were eventually excluded from the analyses between* IFI16-BCL6* and* IFI16-IRF4*. In fact, based on our analysis,* IFI16* expression was suppressed by other molecules in plasma cells, making them not suitable for an appropriate evaluation of the relationship between* IFI16* and* BCL6* or between* IFI16* and* IRF4*.

**Figure 6:**
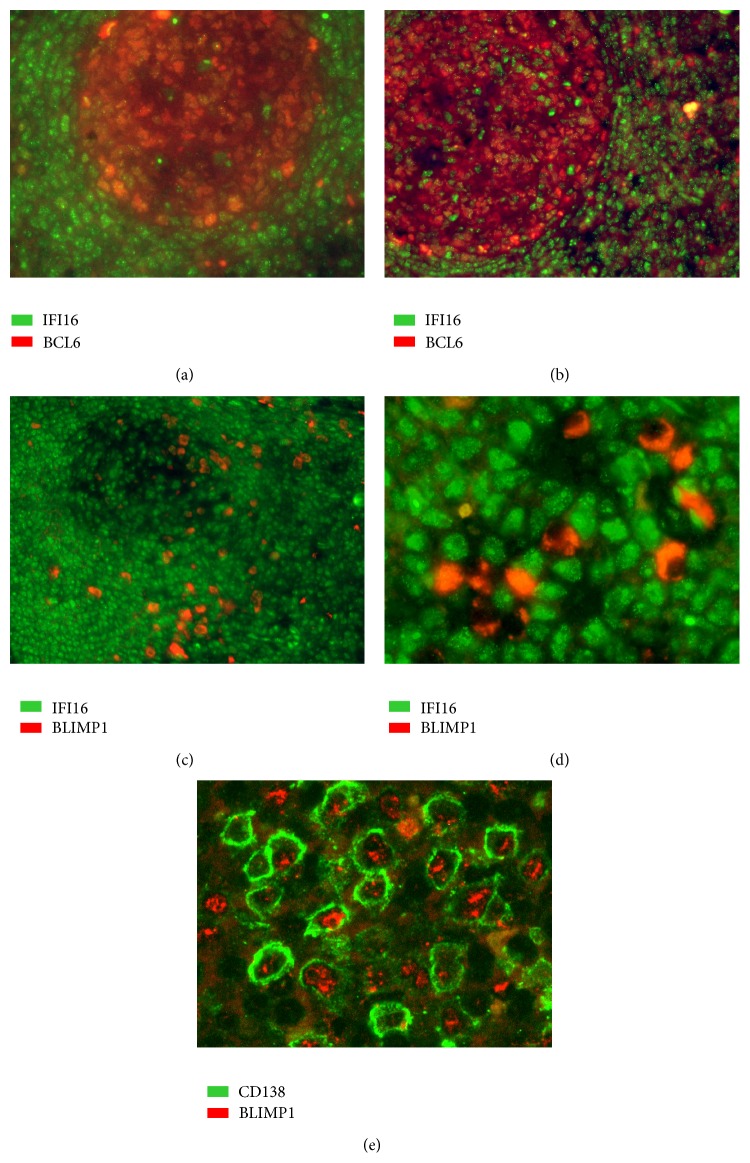
Double-staining immunofluorescence analysis of* IFI16/BCL-6* and* IFI16/BLIMP1* protein expression levels. Double-staining immunofluorescence analysis demonstrated the inverse relationship between IFI16 (green) and either BCL6 (red; (a-b) magnification 200x and 100x, resp.), or BLIMP1 (red; (c-d), magnification 100x and 400x, resp.). The latter, in particular, showed a mutually exclusive expression patterns with IFI16. Specifically, plasma cells in panel (d) were BLIMP1+/IFI16−, while the surrounding lymphocytes were BLIMP1−/IFI16+. In (e) (magnification 400x) double-staining immunofluorescence analysis showed the coexpression of BLIMP1 (red) and plasma cell marker CD138 (green). These micrographs were obtained using an Olympus BX61 microscope equipped with an Olympus DP-70 digital camera; image acquisition, evaluation, and color balance were performed using Cell^∧^F software.

The observation of different expression levels of IFI16 in B-cell subsets and, in particular, the significant* IFI16* mRNA decrease in the crucial GC B-cell subset prompted us to analyse whether IFI16 is regulated by major transcription factors involved in B-cell regulation and differentiation. We considered the transcription factors that are more strictly involved in the modulation of GC cells, including BLIMP1, BCL6, MTA3, PAX5, IRF4, IRF8, XBP1, NF-*κ*B, POU2AF1, Ets family members (as SPIB), BACH2, STAT3, STAT5A, and STAT5B (for details on their expression levels in B-lymphoid subpopulations, see Figure 4). First, we calculated the linear regression coefficient of correlation (coefficient of determination, *R* ^2^) between the expression levels of* IFI16* versus the different transcription factors and selected only those transcription factors with *R* ^2^ > 0.5. Second, we calculated the Pearson correlation and filtered only those with a significant correlation and a* p* value < 0.01. We found that* IFI16* mRNA levels inversely correlated with* XBP1*,* POU2AF1*,* BLIMP1*, and* BCL6* expression, whereas* IRF4*,* STAT3*,* REL*,* SPIB*,* RELA*,* RELB*,* STAT5B,* and* STAT5A* showed a direct correlation with* IFI16* expression.* MTA3*,* PAX5*,* IRF8,* and* BACH2* did not show any significant correlation (Table 2; Figure 5). To validate this observation, we performed double immunostaining assays for IFI16/BCL6 or IFI16/BLIMP1 in a series of reactive lymphoid tissues. Of note, immunostaining confirmed the inverse relationship between IFI16 and BLIMP1 and between IFI16 and BCL6 (Figure 6).

**Figure 7:**
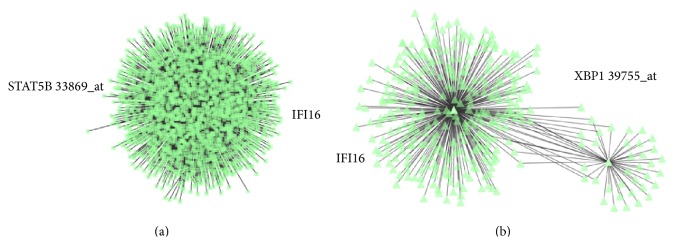
Relationship between* IFI16* and select transcription factors as defined by ARACNe. Examples of direct interaction ((a) STAT5b-IFI16) and indirect interaction ((b) XBP1-IFI16) are plotted. In each panel, the green triangles represent molecules interacting with IFI16 (chosen as the centroid of the analysis).

To assess whether the identified transcription factors could directly target the IFI16 gene, we used a recently developed bioinformatic algorithm (ARACNe), suitable to identify transcriptional targets [9, 49]. Interestingly, ARACNe indicated* IFI16* as a possible direct target of BCL6, RELB, STAT5B, and POU2AF1 but not of BLIMP1, XBP1, IRF4, STAT3, REL, SPIB, RELA, and STAT5A (Table 2; Figure 7). Notably, these results were consistent with those reported by Basso and coworkers, who studied the BCL6 transcriptional network [51]. Altogether, these data indicated that IFI16 modulation during mature B-cell differentiation was strictly associated with the function of different transcription factors. Particularly, IFI16 downregulation during the germinal center (GC) transition appeared to be due to BCL6-mediated transcriptional repression. In contrast, in both naïve and memory B-cells following B-cell receptor (BCR) and CD40 stimulation, NF-*κ*B and STATs protein activation were related to IFI16 levels along with* BCL6* downregulation. Finally, in plasma cells (PCs), the combined effects of BLIMP1, POU2AF1, and XBP1 are likely to maintain the lowest levels of* IFI16* gene expression.

### 3.3. *IFI16* Expression Is Correlated to NF-*κ*B Activation in Some B-Cell Subsets

**Figure 8:**
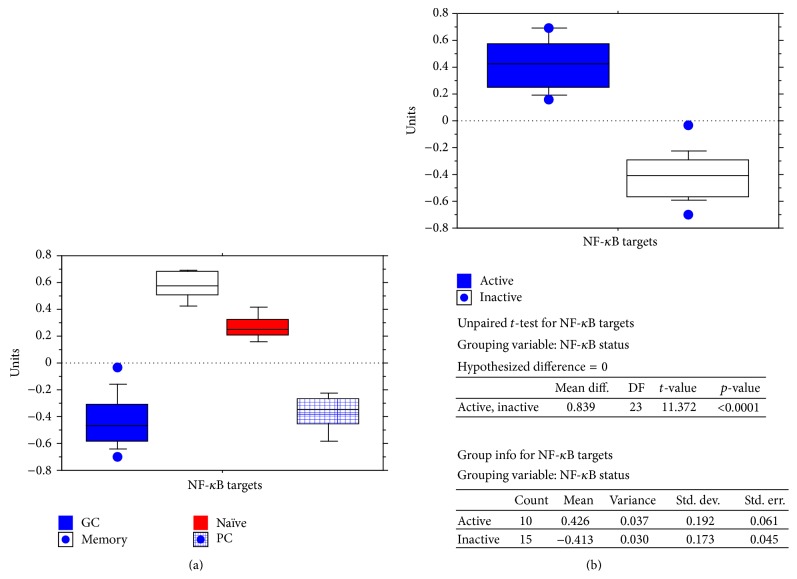
Correlation between* IFI16* and NF-*κ*B target gene expression in normal B-cell subsets. Expression of NF-*κ*B target genes was studied in the different B-cell populations (a). A mean of the normalized expression values of the considered target genes was calculated and defined as the NF-*κ*B signature. Cases with NF-*κ*B signature values below 0 were considered to have an inactive pathway; cases with values above 0 were considered as having an active pathway.* IFI16* mRNA expression levels were then analyzed in the two groups (b). Significantly higher* IFI16* mRNA levels were detected in normal samples with an active NF-*κ*B signature (*p* < 0.0001). Specifically, germinal center cells and plasma cells (low NF-*κ*B signature) were compared to naïve and memory cells (high NF-*κ*B signature).

The NF-*κ*B pathway is involved in B-cell biology and differentiation and some reports have shown that IFI16 can upregulate NF-*κ*B activity in endothelial cells [58, 59]. Therefore, based on the evidence of a correlation between IFI16 and RELA, RELB, and REL, we further analysed this possible interaction in B-cell subsets. Because the functionality of NF-*κ*B subunits (RELA, RELB, and REL) is quite complex and cannot simply be evaluated by detecting changes in their gene expression, we also analyzed the activity of the NF-*κ*B pathway by studying the expression of NF-*κ*B target genes, as reported previously [60]. Higher* IFI16* levels were found in the presence of NF-*κ*B activation (*p* < 0.0001; Table 3; Figure 8), confirming the correlation suggested by the transcriptional levels of the three REL family molecules.

As the relationship between IFI16 and NF-*κ*B is not yet clear, except in endothelial cells, we studied the effects of CD40 (a well-known NF-*κ*B activator in human B-cells) signalling induction on IFI16 levels to determine whether IFI16 is an upstream activator or a downstream target of NF-*κ*B. To do so, we analyzed GEP data that were originally generated in Burkitt lymphoma (BL) cell lines in which CD40 signalling was induced by viral transduction to evaluate the early events after CD40 stimulation [47] (see Supplementary Table 1 in Supplementary Material available online at http://dx.doi.org/10.1155/2015/747645). Although we observed clear activation of NF-*κ*B with increased expression of different NF-*κ*B molecules (including NFKB2 and* NFKBIA*) and NF-*κ*B transcriptional targets (including* ICAM1*,* TRAF1*,* CFLAR*,* IRF4,* and* TNFAIP3*) [61–64], we did not find significant modulation of the* IFI16* gene (*p* = 0.8; fold change, 1.04).

Together, these results indicate a significant association between NF-*κ*B activity and IFI16 expression but apparently excluded a direct effect of NF-*κ*B on IFI16 expression. Therefore, our data support the concept that IFI16 can regulate NF-*κ*B in human B-cells, as already demonstrated in endothelial cells [58, 59].

## 4. Discussion

**Figure 9:**
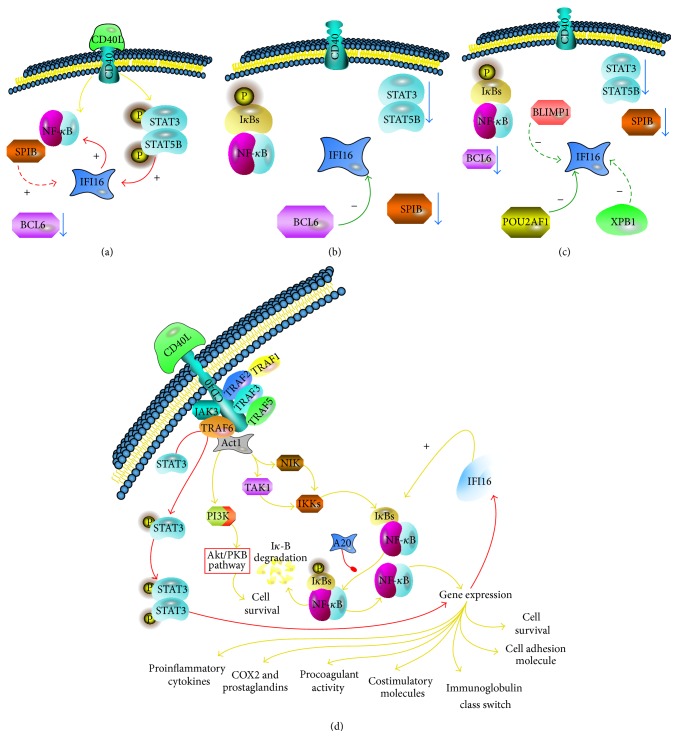
Hypothetical regulation of* IFI16* during mature B-cell differentiation based on the present study. Interactions in naïve and memory (a), germinal center (b), and plasma cells (c) are described. In panel (d), details regarding the effects of CD40 signaling on IFI16 are depicted. Solid arrows indicate possible direct effects while dashed arrows indicate possible indirect effects.

In this paper, we have analyzed* IFI16* mRNA and protein expression levels in normal B-cell subsets and investigated the correlations between levels of* IFI16* and the expression of transcription factors known to play a relevant role in B-cell physiology and differentiation. Specifically, we used GEP analysis in B-cell subsets purified from lymph nodes to explore the transition from naïve to either memory or PCs throughout the GC reaction. Overall, GEP analysis showed that changes in* IFI16* expression were related to cell differentiation stages. The passage from naïve B-cell subsets to proliferating GC cells was associated with a significant downregulation of* IFI16* expression.* IFI16* mRNA levels also declined dramatically when GC cells differentiated into PCs, whereas the shift from GC cells to memory cells demonstrated an increase in IFI16 expression that was comparable to those observed in naïve B-cell subsets. Because* IFI16* can also be regulated posttranscriptionally [57], we evaluated IFI16 protein expression and confirmed the results of the GEP analysis. Immunofluorescence staining of IFI16 in lymph nodes showed a clear decrease in fluorescence in germinal center CD20^+^ B-cells with respect to CD20^+^ B-cells in the mantle zones. These observations indicate that IFI16 expression is regulated in GC cells and its modulation correlated with B-cell differentiation fate. To gain further insights into the control of* IFI16* expression in GC cells, we compared* IFI16* expression with the expression of pivotal transcription factors involved in GC biology and differentiation. GEP analysis showed that* IFI16* expression was significantly related to the expression of the transcription factor* BCL6*. This observation was confirmed through bioinformatic inference of the IFI16 regulatory network using ARACNe, which indicated a direct relationship between* BCL6* and* IFI16*. Moreover, double immunofluorescence analysis indicated low IFI16 protein expression when high BCL6 levels were detected in B-cell compartments. Consistently, a recent study indicated that IFI16 belongs to the first neighborhood of BCL6, within its transcriptional network [51]. BCL6 is a nuclear phosphoprotein that is specifically expressed in the GC in the B-cell lineage and is detectable in the centroblasts and in the majority of centrocytes. BCL6 is a master regulator of GC constitution [4], and BCL6-deficient mice have normal B-cell development but no GC formation [5, 7]. The deregulated expression of* BCL6* induced an increase in GC formation in transgenic mice [7]. In GC, BCL6 regulates differentiation, apoptosis, genotoxic stress response, and cell cycle progression [48, 65–70]. Specifically, BCL6 upregulates proliferation of GCs by suppressing the expression and/or the activation of genes involved in negative cell-cycle regulation such as* TP53*,* PIAS2*, and* p21* [71]. In addition, BCL6 supports the DNA breaks induced by SHM and CSR, suppressing the sensing of DNA damage by* ATM* and* RAD3* inhibition [4, 65]. Conversely, IFI16 downregulates cell proliferation and reinforces genotoxic stress responses by binding several molecular partners including p53 [20, 29, 72–74]. Therefore, it is conceivable that IFI16 transcription inhibition, which is directly controlled by BCL6 during the GC transition, may be relevant to the correct control of cell cycle progression and DNA damage responses in GC cells. In support of this hypothesis, IFI16 acts as a DNA sensor that activates genes involved in cell cycle inhibition and DNA repair [29, 75]. Moreover, IFI16 might act as a scaffold protein that is generally associated with the suppression of cell proliferation by causing cell death or by triggering senescence [21, 23, 26, 27, 32, 76]. We showed evidence that, during GC transition, POU2AF1 could negatively regulated IFI16 as well. On the other hand, no correlation was observed with other transcription factors that play a major role in GC formation [10, 77–79], such as BACH2 and IRF8. Conversely, SPIB, STAT3, and STAT5A showed a positive correlation, supporting the hypothesis that they may contribute to IFI16 expression in naïve and memory cells (Figure 9). Interestingly, a relationship between STAT3 activation and* IFI16* expression during cell apoptosis was detected in medullary thyroid carcinoma and mammary epithelial cells. This finding indicates the possible existence of a more general regulatory network between STAT3 and IFI16 [80, 81]. Notably, by using ARACNe, a robust bioinformatic algorithm [48, 49, 51], we have defined a possible type of relationship between* IFI16* and other molecules. In particular, we have shown evidence of a direct interaction between IFI16 and either BCL6, STAT5B, POU2AF1, or RELB and an indirect relationship (i.e., mediated by intermediate factors) for STAT3, XBP1, REL, SPIB, BLIMP1, RELA, and STAT5A. Interestingly,* IFI16* gene expression after the GC transition was regulated differently in PCs and memory B-cells. In fact,* IFI16* was strongly downregulated in PCs and upregulated in memory B-cells. These two B-cell subsets originate from GC centrocytes cells upon BCR-driven signaling activation [82]. Although* BCL6* is shut off in both cases, the terminal differentiation into plasma cells also requires* PAX5* inactivation and the induction of the transcriptional repressor* BLIMP1* [4, 82]. BLIMP1 has been recognized as a pivotal regulator of the transition between GC and plasma cells. In fact, mice with a B-cell-specific deletion of PRDM1, which is the gene encoding BLIMP1, do not generate PCs [8], and transient transfection experiments with BLIMP1 expression vectors can determine plasmablast differentiation [83].

By testing the possible connection between* IFI16* and* BLIMP1* genes, we found a strong inverse correlation at the gene expression level. Accordingly, we documented their mutually exclusive expression through double-staining immunofluorescence assays. However, ARACNe analysis failed to identify a direct link between* IFI16* and* BLIMP1*, suggesting that this relationship is not direct but is likely mediated by an additional factor. In PCs, our data indicate the possible relevance of XBP1 and POU2AF1 in repressing* IFI16* expression. In particular, POU2AF1 showed a very strong correlation with IFI16 and was indicated by ARACNe analysis as directly interacting with IFI16. In parallel, the analysis of other factors such as MTA-3 and PAX5 that are involved in the regulation of GC differentiation to PCs or memory B-cell subsets did not show any significant association. These results may suggest that* IFI16* mRNA levels are sustained by SPIB and STATs proteins, whereas BCL6 is inhibited, and XBP1, POU2AF1, and BLIMP1 are absent. In contrast,* IFI16* expression is negatively regulated during the transition to GC and during differentiation to PCs by means of a few major negative regulators such as BCL6, XBP1, POU2AF1, and BLIMP1. This particular regulation of* IFI16* expression in PCs may have some interesting parallels with myeloid progenitor-derived cell lineages. IFI16 is expressed in CD34+ hematopoietic progenitors and disappears when the cells differentiate to megakaryocytes and erythrocytes [14, 17]. These observations indicate that IFI16 is tightly regulated in blood cell lineages and plays a regulatory role during the early step of progenitor cell differentiation; however, IFI16 is lost in some lineages, especially when these cells reach their terminal differentiation.

We have also investigated the functional relationship between IFI16 and NF-*κ*B. NF-*κ*B is a transcription factor that is involved in cell survival and activation of B-lymphocytes [47, 84]. Its activation is particularly evident upon B-cell activation, which results in the translocation of active NF-*κ*B into the nucleus where it regulates target genes. NF-*κ*B activation is particularly detectable after B-cell activation, which results in the translocation of active NF-*κ*B into the nucleus where it regulates target genes. Using GEP analysis, we observed that significantly higher* IFI16* gene expression levels accompanied higher NF-*κ*B signature. In GCs and PCs, both* IFI16* mRNA levels and NF-*κ*B signaling decreased, whereas* IFI16* mRNA levels and NF-*κ*B signaling were restored in naïve and memory cells. In the latter two cell subsets, NF-*κ*B activation is sustained, at least in part, by BCR and CD40 signaling. Therefore, we studied the effects of the CD40 downstream cascade on* IFI16* mRNA levels in human B-cells to assess whether NF-*κ*B activation could directly induce changes in IFI16 expression. Despite clear evidence of NF-*κ*B induction as a result of CD40 stimulation, no modification of IFI16 levels was observed. Importantly, consistent with our aim, the experiment was originally designed to detect the early events of CD40 stimulation. Therefore, indirect effects (such as those depending on the subsequent activation of* IRF4* and consequent* BCL6* downregulation) were not observed.

Overall, these results indicated that IFI16 might modulate NF-*κ*B in B-lymphocytes but not* vice versa*. Nonetheless, as our results are based on gene expression modulation, we cannot exclude a more complex functional interaction at the protein level. These data are in line with previous studies demonstrating that IFI16 positively regulates NF-*κ*B activation in endothelial cells [58, 59, 64, 85–87]. IFI16 over expression, indeed, in HUVEC endothelial cell model, triggers the NF-*κ*B complex activation through the inhibition of I*κ*B*α* transcription and expression [58]. In addition, the study of IFI16 activity as DNA sensor indicated that viral and bacterial DNAs activated IFI16 via HIN domains, independently by TLR regulation [39, 75]. IFI16 sensed the presence of viral or bacterial DNA and restricted viral replication of several viruses including HSV, HCMV, EBV, and HIV [40, 88, 89]. In this experimental context [40, 75], it has been determined that IFI16 interacts with STING to modulate positively NF-*κ*B and, then, induce innate response genes [40, 75, 88] suggesting a complex relationship between IFI16 and NF-*κ*B.

## 5. Conclusions

This paper is the first to describe the IFI16 expression pattern in normal human B-cells at the mRNA and protein levels. Although further studies are required to investigate the specific functions of IFI16 in B-cells, the detection of significant changes in IFI16 expression during differentiation stages along with its interaction with several transcription factors, including NF-*κ*B and BCL6, involved in the B-cell biology, suggest an important role of IFI16 in this cell model.

## Supplementary Material

Table S1: Genes differentially expressed upon CD40 triggering.

## Figures and Tables

**(a):** 

B-cell subset	Count	*IFI16* Mean^*∗*^	Standard deviation	Standard error
Naïve cells	5	0.485	0.065	0.029
GC cells	10	0.043	0.231	0.073
Memory cells	5	0.437	0.282	0.126
Plasma cells	5	−2.121	0.409	0.183

**(b):** 

B-cell subset	*p* value	Statistical significance
GC versus memory	0.0131	S
GC versus naïve	0.0062	S
GC versus plasma cells	<0.0001	S
Memory versus naïve	0.78	NS
Memory versus plasma cells	<0.0001	S
Naïve versus plasma cells	<0.0001	S

^*∗*^Normalized gene expression value.

**Table 2:** Relation of *IFI16* with the main transcription factors regulating B-cell fate in terms of correlation, regression, and mutual information (in bold, genes with significant correlation and regression values).

Gene	*R*2	Pearson correlation	*p* value	Mutual information *p* value 0.01
**STAT3**	**0.928**	**0.963**	1.22**E** − 14	**0.06422802**
**XBP1**	**0.829**	**−0.91**	2.65**E** − 10	**0.081934884**
**REL**	**0.807**	**0.898**	1.11**E** − 09	
**SPIB**	**0.795**	**0.892**	2.22**E** − 09	
**BLIMP1**	**0.722**	**−0.85**	7.65**E** − 08	
**RELA**	**0.718**	**0.847**	9.27**E** − 08	
**RELB**	**0.708**	**0.841**	1.38**E** − 07	**0.10159171**
**POU2AF1**	**0.636**	**−0.797**	1.89**E** − 06	**0.12605515**
**IRF4**	**0.63**	**0.794**	**0.03**	
**STAT5B**	**0.629**	**0.793**	2.26**E** − 06	**0.09627818**
**STAT5A**	**0.594**	**0.77**	6.57**E** − 06	
**BCL6**	**0.51**	**−0.714**	**0.0004**	
BACH2	0.49	0.7	9.90*E* − 05	
IRF8	0.219	0.539	0.005	
PAX5	0.093	0.305	0.138	
MTA3	8.90*E* − 05	−0.009	0.05	

**Table 3:** *IFI16* expression according to NF-*κ*B activation status.

		NF-*κ*B status	
		Active	Inactive
*IFI16* expression	Mean	0.426	−0.413
	Mean difference	0.839	
	Variance	0.037	0.03
	Std. dev.	0.192	0.173
	Std. err.	0.061	0.045

	*p* value	<0.0001	
