# Supplementary material for: IFI16 Expression Is Related to Selected Transcription Factors during B-Cell Differentiation
Source: J Immunol Res. 2015 Jun 22;2015:747645. doi: 10.1155/2015/747645 (PMC4491573; doi:10.1155/2015/747645)
Supplement: Supplementary file 1 — Table S1: Genes differentially expressed upon CD40 triggering. [file 747645.f1.pdf]

Supplementary Table 1. Genes differentially expressed upon CD40 triggering ($p<0.05$; fold change ≥2; false discovery rate according to Benjamini-Hockeberg)

Probe Set ID	Gene Symbol	p (Corr)	FC (abs)	Regulation CD40neg vs CD40pos
31472_s_at	CD44	7,86E-05	2,4346426	down
31557_at	TMSB4X	6,47E-08	3,2688901	down
33513_at	SLAMF1	8,82E-09	6,1727204	down
35000_at	TNFSF9	1,86E-04	2,2321618	down
35957_at	SNN	7,85E-05	2,0018954	down
36239_at	POU2AF1	1,41E-04	2,5836744	up
36296_at	LTA	1,39E-06	4,3666887	down
36773_f_at	HLA-DQB1 /// LOC100133583 /// LOC100293977	2,51E-06	5,227808	down
36808_at	PTPN22	9,64E-04	2,0405164	up
37136_at	SH2B2	2,31E-05	2,113745	up
37420_i_at	HLA-F	1,59E-06	3,0793452	down
37455_at	CXCR5	3,13E-07	2,7838635	down
37483_at	HDAC9	8,25E-04	2,3922713	up
38242_at	BLNK	1,49E-04	2,0941443	up
38578_at	CD27	1,76E-07	3,1569505	up
38893_at	NCF4	5,93E-05	2,001027	up
38894_g_at	NCF4	4,24E-06	2,0301292	up
38917_at	TRD@	0,001972864	2,0782807	up
39231_at	CHD1	8,29E-06	2,308072	down
40362_at	NFKB2	1,33E-04	2,2769434	down
41106_at	KCNN4	2,53E-06	2,1241715	down
41690_at	ARID5B	5,86E-07	2,946656	down
31856_at	LRRC32	1,04E-05	3,483104	down
32129_at	RNF115	3,69E-05	2,0368357	down
32640_at	ICAM1	9,25E-05	2,6988692	down
33804_at	PTK2B	5,71E-05	2,1969037	down
34677_f_at	LOC162632 /// LOC220594 /// USP32 /// USP6	9,98E-04	2,1406088	up
35614_at	TCFL5	6,83E-05	3,2490892	down
35985_at	AKAP2 /// PALM2-AKAP2	8,72E-05	2,2250805	up
36537_at	ARHGEF18	1,29E-05	2,7902102	up
36878_f_at	HLA-DQB1	1,35E-06	5,153037	down
37177_at	CD58	9,80E-09	9,740763	down
37536_at	CD83	1,13E-06	3,6934023	down
37542_at	LHFPL2	2,94E-06	4,9178615	down
37625_at	IRF4	2,71E-05	2,4647584	down
38276_at	NFKBIE	8,56E-07	2,1693919	down
38631_at	TNFAIP2	1,61E-08	5,8500648	down
38717_at	METTL7A	5,55E-05	2,6242304	up
39039_s_at	UBE2J1	4,39E-04	2,1238678	up
39040_at	UBE2J1	3,39E-05	2,479652	up
39402_at	IL1B	1,93E-05	2,549196	down
39728_at	IFI30	1,71E-06	2,0604205	down
40091_at	BCL6	2,17E-05	4,650867	up

40153_at	TAP1	2,38E-06	2,454961	down
41723_s_at	HLA-DRB1 /// HLA-DRB3 /// HLA-DRB4 /// HLA-DRB5 /// HLA-DRB6 /// LOC100294036 /// LOC100509582 /// LOC100510495 /// LOC100510519	1,51E-04	2,3210213	down
32193_at	PLXNC1	2,56E-05	2,0570738	down
32227_at	SRGN	5,57E-07	5,2068543	down
32773_at	HLA-DQA1	1,59E-05	2,2090333	down
32786_at	JUNB	5,81E-05	2,0716155	down
33338_at	STAT1	1,51E-04	2,2681913	down
33439_at	SIK1	2,26E-05	2,621589	down
34885_at	SYNGR2	1,24E-07	2,6065996	down
36103_at	CCL3	1,63E-07	11,555569	down
36179_at	MAPKAPK2	1,66E-05	2,345878	down
36180_s_at	MAPKAPK2	2,14E-05	2,0059936	down
36575_at	RGS1	2,64E-07	4,463683	down
36617_at	ID1	2,93E-05	2,7934341	up
36618_g_at	ID1	1,23E-04	2,735503	up
37006_at	IGJ	1,13E-04	2,590326	up
37024_at	LITAF	2,67E-07	8,536645	down
37025_at	LITAF	1,00E-05	2,7826881	down
37043_at	ID3	3,33E-07	2,1811166	up
37328_at	PLEK	2,66E-06	3,2922337	down
37691_at	LOC729991-MEF2B /// MEF2B	3,07E-06	2,289475	up
37711_at	MEF2C	5,25E-04	2,202418	down
37759_at	LAPTM5	1,06E-04	4,201958	down
38096_f_at	HLA-DPB1	0,004603755	2,1534047	down
38391_at	CAPG	4,27E-06	2,1796553	down
38415_at	PTP4A2	0,001873488	2,2029583	up
39822_s_at	GADD45B	1,29E-05	2,27891	down
39827_at	DDIT4	1,67E-04	3,127329	down
41790_at	ALDH5A1	1,02E-04	2,0964756	up
32616_at	LYN	4,24E-06	2,3925354	down
2042_s_at	MYB	5,15E-07	2,3905349	down
2036_s_at	CD44	2,50E-05	3,9111266	down
2024_s_at	LYN	5,90E-05	2,1390023	down
2002_s_at	BCL2A1	2,46E-06	3,6291728	down
2011_s_at	BIK	5,65E-04	2,3138027	up
1909_at	BCL2	2,98E-05	2,3839056	down
1867_at	CFLAR	1,62E-05	2,2425609	down
1868_g_at	CFLAR	1,13E-06	3,061496	down
1852_at	TNF	1,90E-08	3,9721708	down
1857_at	SMAD7	3,10E-05	2,425706	down
1520_s_at	IL1B	1,72E-05	2,4006371	down
1461_at	NFKBIA	2,44E-06	2,746719	down
1402_at	LYN	1,82E-04	2,1747775	down
1373_at	TCF3	1,27E-05	2,8388565	up
1292_at	DUSP2	2,83E-07	3,3349469	down
1126_s_at	CD44	2,03E-05	3,64973	down
1097_s_at	CCR7	8,16E-07	8,9604025	down
1110_at	TRD@	1,81E-05	2,0116029	up

1003_s_at	CXCR5	2,38E-06	2,2997074	down
1004_at	CXCR5	1,22E-07	3,6967244	down
925_at	IFI30	5,35E-05	2,2930064	down
931_at	GPR183	8,13E-07	7,246555	down
848_at	TRAF1	4,17E-07	3,7861829	down
849_g_at	TRAF1	5,32E-09	4,969944	down
717_at	TRIB2	9,65E-05	2,185458	up
595_at	TNFAIP3	1,59E-07	3,632786	down
544_at	NFKB2	3,57E-05	2,3456557	down
545_g_at	NFKB2	1,94E-05	2,1398354	down
535_s_at	NFKB2	0,001768874	2,1464076	down
506_s_at	STAT5A	1,22E-07	3,8831184	down
478_g_at	IRF5	2,70E-06	2,313924	down
258_at	LTA	1,93E-05	3,319018	down
1456_s_at	IFI16	0,85058695	1,0393113	up
